# Secretomes derived from osteogenically differentiated jaw periosteal cells inhibit phenotypic and functional maturation of CD14^+^ monocyte-derived dendritic cells

**DOI:** 10.3389/fimmu.2022.1024509

**Published:** 2023-01-09

**Authors:** Wanjing Cen, Felix Umrath, António José Salgado, Siegmar Reinert, Dorothea Alexander

**Affiliations:** ^1^ Department of Oral and Maxillofacial Surgery, University Hospital Tübingen, Tübingen, Germany; ^2^ Clinic for Orthopaedic Surgery, University Hospital Tübingen, Tübingen, Germany; ^3^ Life and Health Sciences Research Institute (ICVS), School of Medicine, University of Minho, Braga, Portugal; ^4^ ICVS/3B’s-PT Government Associate Laboratory, University of Minho, Braga, Portugal

**Keywords:** mesenchymal stem cells, jaw periosteal cells, osteogenic differentiation, secretomes, CD14+ monocytes, dendritic cell maturation, dextran-uptake, mixed lymphocyte reactions

## Abstract

The jaw periosteal tissue is generally recognized as a suitable source for the isolation of mesenchymal stem cells (MSCs). In previous studies we showed evidence that two- and three-dimensionally cultured jaw periosteum-derived MSCs (JPCs) are able to induce a more immature phenotype of dendritic cells (DCs). To further expand our knowledge of JPCs’ immunoregulative function, we investigated the effects of JPC secretomes derived from undifferentiated (CO) or osteogenically differentiated cells (treated with or without dexamethasone: OB+/-D) on CD14^+^ monocyte-derived DCs (MoDCs). We detected a remarkably reduced formation of MoDC homotypic clusters under the influence of secretomes from osteogenically induced JPCs. Further, significantly decreased numbers of CD83^+^ cells, up-regulated CD209 and down-regulated CD80, CD86 and CD197 expression levels were detected on the surface of MoDCs. Whereas secretomes from JPCs osteogenically stimulated with dexamethasone significantly enhanced FITC-dextran uptake capacity of MoDCs, the increase by secretomes of JPCs treated without dexamethasone did not reach significance. The analysis of mixed lymphocyte reactions revealed that OB+/-D secretomes were able to significantly reduce the numbers of proliferating CD14^-^ peripheral blood mononuclear cells (PBMCs) and of proliferating CD4^+^ T cells. The OB-D secretome significantly promoted the expansion of regulatory CD25^+^ T cells. Regarding gene expression of MoDCs, remarkably up-regulated mRNA expression of CD209, HLA-DRA, CSF3, IL10 and IL8 was detected when DCs were cultured in the presence of OB+/-D secretomes. At the same time, secretomes seemed to have an impact in the down-regulation of IFNγ and IL12B gene expression. At protein level, OB+/-D secretomes significantly up-regulated IL-10 and IDO (indoleamine-pyrrole 2,3-dioxygenase) levels whereas IL-12/IL-23p40 levels were down-regulated in supernatants of MoDCs when cultured under the presence of OB+/-D secretomes. Taken together, while secretomes from untreated JPCs had only little effects on the process of maturation of MoDCs, secretomes derived from osteogenically induced JPCs were able to inhibit the phenotypic and functional maturation of MoDCs.

## 1 Introduction

Mesenchymal stem cells (MSC) are guardians of adult tissue maintenance and generally considered to be weakly immunogenic cells which implement their immunomodulatory functions by producing a plethora of growth factors, cytokines and chemokines (1, 2). Bone marrow MSCs suppress different T, B, NK and dendritic cell (DC) functions involved in their maturation, activation and antigen presentation process (1).

Jaw periosteum-derived cells (JPCs) with their advantages of simple tissue harvesting and cell isolation, represent a highly suitable mesenchymal stem cell source for regeneration purposes in the jaw bone area. In different studies we were able to prove the osteogenic potential of these cells (3–5). The primary function of the immune system is to protect the host from foreign pathogens, but its dysregulation can lead to failure of transplanted organs or in the case of tissue engineering (TE) of implanted TE constructs. Reducing the immune response to implanted cell-seeded constructs may be achieved by choosing a cell source that can prevent activation of the immune system *via* suppressing the function of antigen-presenting cells (APC). In previous studies, we started with examinations in terms of the immunogenicity of JPCs and could demonstrate that 2D/3D cultured JPCs suppress monocytic DC maturation in a transwell coculture system (6, 7) and that they are also empowered to regulate THP-1-derived macrophage polarization in a direct and a horizontal coculture in system (8, 9).

DCs are rare, heterogeneous bone marrow (BM)-derived professional APCs (10), which arise from hematopoietic stem cells through specialized progenitor subsets (11). They can be broadly categorized as hematopoietic progenitor cell-derived DCs and monocyte-derived DCs. Monocytes are precursors of macrophages, and can also serve as DC precursors (12). Monocyte-derived DCs have been the model for studies of DC development and function (13). DCs build an important link between the innate and adaptive immune system and are crucial in determining the balance between immunity and tolerance (14–16). In the steady state, the majority of DCs are in an immature state within tissues where they constantly present self-antigens to T cells but lack adequate co-stimulatory ability, deliver inhibitory and produce tolerance-promoting cytokines, such as IL-10 (15) and maintain self-tolerance (17–19). Further, immature DCs promote T cell deletion and/or expansion of regulatory T cells (20, 21), inducing peripheral tolerance. When activated, immature DCs recognize danger signals and undergo further maturation (22). Mature DCs lose their antigen-capturing and obtain an antigen-presenting combined with the T cell co-stimulatory ability, thereby migrate in the following step to the lymph nodes, and stimulate T cells (23).

Toward the development of cell-free therapeutic strategies for regenerative medicine, the usage of MSC-sourced secretome receives more and more awareness. Compared to the difficulties associated with the transplantation of living and proliferating cell populations in terms of embolism, transmission of infectious diseases or immune incompatibility, secretomes are safer and dosage and potency/storage can be evaluated in a similar manner to that of pharmaceuticals (24). The aim of the present study was to investigate whether JPC-secreted factors are efficient enough to inhibit DC activation/maturation. Since for bone tissue engineering purposes osteogenically differentiated MSCs are commonly used, we compared the effects emanating from both undifferentiated and osteogenically induced JPCs. Dexamethasone is typically supplemented to the osteogenic differentiation medium of MSCs. However, our previous study has shown that dexamethasone is not indispensable for the osteogenic differentiation of JPCs when the medium was supplemented with human platelet lysate (hPL) instead of fetal calf serum (FCS) (25). Therefore, we analyzed the effects of JPC secretomes from untreated and osteogenically induced cells with and without dexamethasone on the morphology, phenotype and function of MoDCs.

## 2 Materials and methods

### 2.1 Isolation and culture of jaw periosteal cells

JPCs obtained from three donors were included in this study after the approval (number 618/2017BO2) from the local ethics committee. Jaw periosteal tissue was extracted during routine surgery after obtaining written informed consent and JPCs were isolated as mentioned previously (26). JPCs were cultured in DMEM/F-12+GlutaMAX medium (Gibco, Waltham, MA, USA) supplemented with 10% human platelet lysate (hPL, PL BioScience GmbH, Aachen, Germany), 1% penicillin-streptomycin (Pen-Strep, Lonza, Basel, Switzerland) and 1% amphotericin B (Biochrom, Berlin, Germany). In previous works, we published minimal criteria fulfillment and tri-lineage differentiation potential of JPCs (25–27).

### 2.2 Osteogenic differentiation of JPCs

The cell culture flasks and plates were coated with 0.1% gelatin from bovine skin (Sigma-Aldrich, St. Louis, MO, USA) prior to osteogenic differentiation in order to allow better JPC adherence and avoid detachment from the culture flasks during long-term incubation. For osteogenic differentiation, JPCs were cultured in osteogenic medium (DMEM/F-12+GlutaMAX medium, 10% hPL, 1% Pen-Strep, 1% amphotericin B, 100 µM L ascorbic acid 2-phosphate (Sigma-Aldrich, St. Louis, MO, USA), 10 mM β glycerophosphate (AppliChem, Darmstadt, Germany) supplemented with or without 4 µM dexamethasone (Sigma-Aldrich, St. Louis, MO, USA) for 10 days for secretome collection or for 15 days for the analysis of the JPC osteogenic potential.

### 2.3 JPC secretome collection and enrichment

For secretome collection, 0.1% gelatin was used to coat the 175- cm^2^ cell culture flasks and incubate at 37°C for at least 30 min. JPCs of passage 5 were seeded onto the coated cell culture flasks at a cell density of 1 million cells per flask. After overnight incubation, medium was changed to osteogenic medium with (OB+D) or without dexamethasone (OB-D). Cells cultured in normal medium (CO) were used as control. After 10-day of cultivation, cells were washed with PBS (Lonza, Basel, Switzerland) three times and 37 mL DMEM/F12 medium containing 1% Pen-Strep and 1% amphotericin B were added to the JPCs for accurately 24 h. Secretome was collected from the flasks and centrifuged at 600 g to remove cell debris. 34 mL of supernatant were collected and shock frozen in liquid nitrogen instantly and stored at -80°C. After thawing, the secretome was concentrated 100-fold by centrifugation using Vivaspin 20 (Sartorius, Goettingen, Germany) as recommended by the manufacturer. Collected and enriched secretomes (S_CO, S_OB+D, S_OB-D) from three JPCs donors were pooled before use for further experiments. The concentration of JPC secretomes was determined using the Qubit protein assay kit (Invitrogen, Waltham, MA, USA) and the Qubit 3.0 fluorometer (Thermo Fisher Scientific, Waltham, USA) following the manufacturer’s instructions. 100-fold basal DMEM/F12 medium was used as a blank. In **Table 1** the measured concentrations of the 100-fold concentrated JPC secretomes (S_CO, S_OB+D, S_OB-D) are given.

**Table 1:** Protein concentration of 100-fold concentrated JPC secretomes.

		S_CO	S_OB+D	S_OB-D
Concentration (mg/mL)	1^st^ thawing	0.93 ± 0.03	0.92 ± 0.02	1.04 ± 0.07
	2^nd^ thawing	0.92 ± 0.09^#^	0.97 ± 0.05^#^	1.16 ± 0.11^#^

The aliquots of concentrated secretomes derived from JPCs treated with control medium (S_CO), osteogenic medium with or without dexamethasone (S_OB+D/S_OB-D) stored at -80°C were thawed before first use (1^st^ thawing) and stored at -20°C till second use for medium change (2^nd^ thawing). The protein concentration of secretomes was determined using the Qubit protein assay kit and the Qubit 3.0 Fluorometer. 100-fold concentrated DMEM/F12 medium was used as a blank. The data of three independent experiments are shown as means ± SD and compared using paired t test (n=3, ^#^represented no significant difference compared to 1st thawing).

### 2.4 Alizarin red staining and OsteoImage mineralization assay of JPCs

After 15 days of osteogenic stimulation, JPCs were fixed with fixation buffer (Biolegend, California, USA) and stained with 40 mM Alizarin red solution (pH 4.2, Sigma-Aldrich) for 20 min. Unbound dye was removed by washing with deionized water and images were taken using an inverted microscope (Leica, Wetzlar, Germany). The stained plates were quantified using alizarin red S staining quantification assay kit (ScienCell, California, USA) following the manufacturer’s instructions and photometrical quantification of the alizarin staining was performed at a wavelength of 405 nm using a microplate reader (Biotek, Bad Friedrichshall, Germany). JPCs were further stained for hydroxyapatite detection using the OsteoImage mineralization assay kit (Lonza, Basel, Switzerland) following the manufacturer’s instructions. Images were taken using an Axio Observer Z1 fluorescence microscope (Zeiss, Oberkochen, Germany).

### 2.5 Separation of CD14^+^ cells from human peripheral blood mononuclear cells

PBMCs were isolated from fresh blood collected using S-monovettes with 1.6 mg EDTA/mL (SARSTEDT AG & Co. KG, Nümbrecht, Germany) using the gradient centrifugation with Ficoll-Paque PLUS (Cytiva, Uppsala, Sweden). After extraction of the PBMC fraction and washing three times with PBS, PBMCs were used for further separation of the CD14^+^ population using the CD14 MicroBeads kit (Miltenyi Biotec, Bergisch Gladbach, Germany) following the manufacturer’s instructions. The obtained CD14^+^ cells were used for DC differentiation experiments and the fraction of the CD14^-^ cells was cryopreserved in freezing medium consisted of 45% RPMI 1640 medium (Gibco, Waltham, MA, USA), 45% fetal calf serum (FCS, Gibco, Waltham, MA, USA), and 10% DMSO (Sigma-Aldrich, Darmstadt, Germany), and used for further mixed lymphocyte reactions experiments.

### 2.6 Differentiation of CD14^+^ monocyte-derived dendritic cells

CD14^+^ monocytes were seeded at a density of 10^6^ monocytes/mL in 24-well plates and cultured for 6 days in RPMI 1640 medium containing 10% FCS, 1% pen-strep and 1% amphotericin B, supplemented with the DC cytokine cocktail (40 ng/mL IL-4 and 100 ng/mL GM-CSF (Sigma-Aldrich, Darmstadt, Germany) for the first 5 days; 40 ng/mL IL-4, 100 ng/mL GM-CSF, 10 ng/mL TNF- α, 10 ng/mL IL-1β, 10 ng/mL IL-6 (Tebu Bio, Offenbach, Germany), and 1 µg/mL PGE2 (Bio Trend, Köln, Germany) for the last 24 h). Four different DC populations were generated, three of them were treated with 5-fold or 10-fold concentrated JPC secretomes (from untreated JPCs (S_CO), and from osteogenically induced JPCs with (S_OB+D) or without (S_OB-D) dexamethasone stimulation for 10 days). The fourth DC population served as a control sample without additional secretome supplementation (control).

### 2.7 Flow cytometry analysis of cell surface marker expression on MoDCs

CD14^+^ monocytes and the four differentiated DC populations were collected and analyzed for surface marker expression by flow cytometry. 2 × 10^5^ cells per sample were blocked with 20 µL 10% Gamunex (human immune globulin solution, Talecris Biotherapeutics, Germany) in FACS buffer (PBS, 0.1% BSA, and 0.1% sodium azide) for 15 min on ice. Cells were incubated on ice with PE-labeled mouse anti-human CD83 (BD Pharmingen, New Jersey, USA, clone: HB15e, isotype: IgG1κ), CD80 (clone: 2D10, isotype: IgG1κ), CD86 (clone: BU63, isotype: IgG1κ), and CD1a (clone: HI149, isotype: IgG1κ), and APC-labeled mouse anti-human CD209 (clone: 9A9E8, isotype: IgG2aκ), HLA-DR (clone: L243, isotype: IgG2aκ), CD14 (clone: M5E2, isotype: IgG2aκ) and CD197 (clone: G043H7, isotype: IgG2aκ) antibodies (BioLegend, San Diego, USA) for 15 min in dark. For the isotype controls, PE-labeled IgG1 and APC-labeled IgG2a antibodies (Biolegend, San Diego, CA, USA) were used. After washing with FACS buffer twice, cell pellets were resuspended in 200 μL FACS buffer and analyzed using the Guava easyCyte 6HT-2L device (Merck Millipore, Billerica, MA, USA). The analyzed cells were gated according to their size and granularity (**Supplementary Figures 1A, B**). All percentages, mean fluorescent intensities and statistical analysis were performed inside the gate, thus excluding cell debris. For data evaluation, the guavaSoft 2.2.3 (InCyte 2.2.2, Luminex Corporation, Chicago, IL, USA) software was used.

### 2.8 Analysis of the phagocytic activity of generated DC populations

To measure the phagocytotic activity of the four generated DC populations, cells were resuspended in 100 ml RPMI 1640 medium containing 10% FCS and incubated with 1 mg/mL FITC-dextran (wt 40000; Sigma-Aldrich, St. Louis, MO, USA) at 37°C and on ice (negative control) for 60 min. After incubation, cells were washed twice with cold PBS and fixed with fixation buffer (BioLegend, San Diego, USA). The uptake of FITC-dextran by DCs was determined by flow cytometry. The analyzed cells were gated according to their size and granularity (**Supplementary Figure 1C**). All percentages, mean fluorescent intensities and statistical analysis were performed inside the given gate, thus excluding cell debris. At least 5000 cells per sample were analyzed. For confocal microscopy image acquisition, cells were stained for nuclei using DAPI (Life Technologies, Lima, USA) and spun onto a Shandon Cytospin 4 slide (Thermo Fisher Scientific, Waltham, USA). The slides were examined with a confocal laser scanning microscope system (Leica NS-CT; Leica Lasertechnik, Heidelberg, Germany) fitted with lasers emitting light at 488 and 405 nm.

### 2.9 Analysis of T cell stimulatory capacity of generated DC populations

To analyze T cell stimulatory ability of MoDCs generated under the presence of different JPC secretomes, CD14^-^ PBMCs were thawed 1 day before coculturing with DCs. Before use, thawed cells were stained with 1 µg/mL propidium iodide (PI, Invitrogen, Waltham, MA, USA) for cell viability by flow cytometry on the same day (day 0) and the day after thawing (day 1), as shown in **Table 2**. On day 1, CD14^-^ PBMCs were used for further separation of the CD4^+^ T cells using the CD4^+^ T cell isolation kit (Miltenyi Biotec, Bergisch Gladbach, Germany) following the manufacturer’s instructions. The CD4^+^ cell fraction was stained with mouse anti-human CD4-PE (clone: M-T466, isotype: IgG1κ, Miltenyi Biotec, Bergisch Gladbach, Germany) and analyzed using flow cytometry. CD14^-^ PBMCs and CD4^+^ T cells were firstly labeled with the CFSE cell proliferation kit (Invitrogen, Waltham, MA, USA) following manufacturer recommendations respectively. 2 × 10^5^ CFSE-labeled cells were cocultured (ratio of 1:1) with DCs previously generated under the four different conditions (in the absence of JPC secretome or under the addition of the 3 JPC secretomes) in RPMI 1640 medium containing 10% FCS in U-bottom 96-well plates for 72 h. CD14^-^ PBMCs were analyzed by flow cytometry. CD4^+^ T cells were stained with mouse anti-human CD25-APC (clone: 3G10, isotype: IgG1κ, Miltenyi Biotec, Bergisch Gladbach, Germany) and at least 5000 cells per sample were analyzed by flow cytometry. The monocultured CFSE-labeled cells were used as unstimulated control. The analyzed cells were gated according to their size and granularity (**Supplementary Figures 1D–G**). The mean fluorescent intensities and statistical analysis were performed inside the gate, excluding cell debris. For data evaluation, the guavaSoft 2.2.3 software and FlowJ 10 (BD, New Jersey, USA) were used.

**Table 2:** Percentages of viable CD14^-^ PBMCs after thawing.

	Day 0	Day 1
Viable cell percentage (%)	95.64 ± 1.17	92.12 ± 0.97

Frozen CD14^-^ PBMCs were thawed one day before use. Cell viability was measured after propidium iodide staining by flow cytometry on day 0 and the second day (day 1) after thawing. The data of three independent experiments are shown as mean ± SD.

### 2.10 Quantitative gene expression analysis of generated DC populations

The total mRNA of DCs generated under the four different conditions was extracted using the NucleoSpin RNA kit (Macherey-Nagel, Dueren, Germany) as manufacturer recommended. After determining the concentrations of RNA using the Nanodrop One device (Thermo Fisher Scientific, Waltham, USA), 500 µg of RNA was used for cDNA synthesis using the LunaScript RT SuperMix Kit (New England Biolabs, Ipswich, MA, USA). The mRNA expression levels were quantified by the QuantStudio 3.0 device (Thermo Fisher Scientific, Waltham, USA). The LUNA universal probe qPCR master mix (New England Biolabs, Ipswich, MA, USA) and primers of indicated genes (GAPDH (Hs.PT.39a.22214836), CD209 (Hs.PT.58.15573799.g), HLA-DRA (Hs.PT.58.15096946), CCL3 (Hs.PT.58.27485430.g), CSF3 (Hs.PT.58.27044427.g), IL10 (Hs.PT.58.2807216), IL8 (Hs.PT.58.39926886.g), IL4 (Hs.PT.58.46539563.g), TNF (Hs.PT.58.45380900), IFNG (Hs.PT.58.3781960), IL12A (Hs.PT.58.1687020), IL12B (Hs.PT.58.2925830), IL12RB1 (Hs.PT.58.14547172) and IL12RB2 (Hs.PT.58.40444640)) were purchased from Integrated DNA Technologies (Coralville, Iowa, USA) and used for the PCR reactions. PCR amplification of each of the indicated genes was carried out for 40 circles (95°C 60 sec, 95°C 1 sec, and 60°C 20 sec). The relative gene expression levels were calculated using the ^ΔΔ^Ct method and the data presented as 2^-ΔΔCt^.

### 2.11 Analysis of immunomodulatory factors in supernatants of generated DC populations and JPC secretomes

IL-10, IDO (Indoleamine-pyrrole 2,3-dioxygenase) and IL-12/IL-23p40 secretion levels in supernatants of generated DC populations and 5-fold concentrated JPC secretomes were quantified using the human IL-10 (assay range: 0.39-25.0 pg/mL), human IDO (assay range: 0.819-200 ng/mL), and human IL-12/IL-23p40 (assay range: 0.0313-2 ng/mL) ELISA kits (Invitrogen, Waltham, MA, USA) according to the manufacturer’s instructions. All measurements were performed in triplicates and determined at 450 nm with a microplate reader (Biotek, Bad Friedrichshall, Germany).

### 2.12 Statistical analysis

Statistical analysis was conducted for three independent experiments using the GraphPad Prism 8.1.0 software (GraphPad, San Diego, CA, USA). Data were tested for normality using the Shapiro-Wilk test. All results were presented as means ± SD. Results of JPC secretome concentration were compared using paired t test. Results of FACS measurements of cell surface markers on CD14^+^ monocytes and MoDCs (Control group), and ELISAs were compared using the multiple t test, other results were compared using one-way ANOVA followed by Tukey’s multiple comparisons tests. P values < 0.05 were considered significant.

## 3 Results

### 3.1 Analysis of the osteogenic potential of JPCs

**Figure 1:**
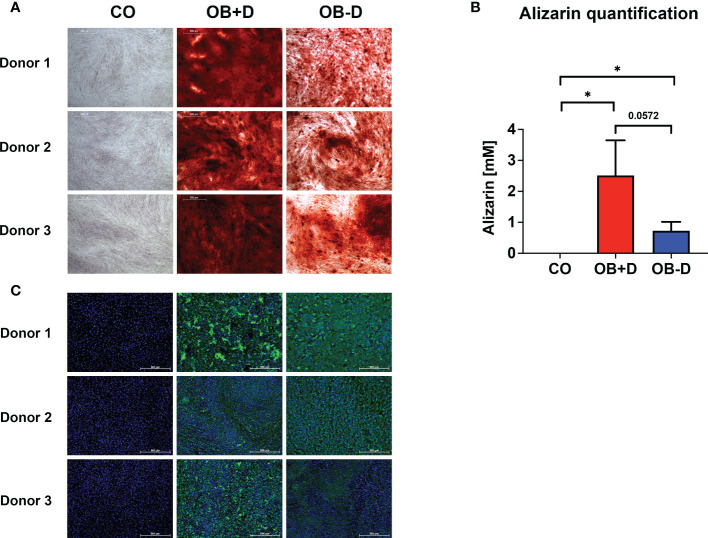
Mineralization capacity of JPCs from used donors. JPCs (3 donors) were cultured with control medium (CO), osteogenic medium with (OB+D) and without dexamethasone (OB-D) for 15 days. **(A)** Calcium deposits were stained with alizarin red. **(B)** Quantification of alizarin staining. Data of three independent experiments are given as means ± SD and compared with each other using student’s t test (n=3, *p < 0.05). **(C)** Hydroxyapatite particles were stained using the fluorescent OsteoImage assay (green) and cell nuclei were stained with Hoechst (blue). Scale bars represent 500 µm.

The three used JPC donors mineralized in both media (with/without dexamethasone, OB+/-D) containing 10% hPL after 15 days of osteogenic induction. JPCs cultured in the OB-D medium revealed less calcium deposits as stained by alizarin red (**Figure 1A**). The differences between the mineralization in osteogenically stimulated JPCs (OB+/-D) and control wells (CO) were significant. However, differences between OB+D and OB-D conditions were not statistically significant, due to high donor variations (**Figure 1B**). Consistent with the results of the alizarin red staining, hydroxyapatite particles of smaller size were detected under OB-D condition by the fluorescent OsteoImage staining (**Figure 1C**).

### 3.2 Effects of JPC secretomes on cell surface marker expression of MoDCs

**Figure 2:**
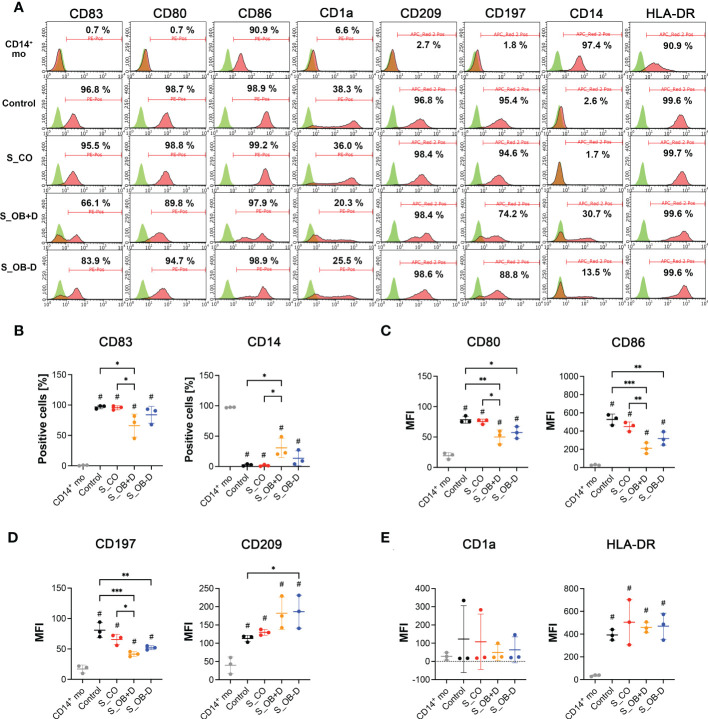
Cell surface marker expression of CD14^+^ monocytes and DCs generated by various treatments. **(A)** Flow cytometry analysis (FACS) of cell surface markers on CD14^+^ monocytes (CD14^+^ mo) and DCs generated without (control) or in the presence of JPC secretomes derived from untreated (S_CO), and osteogenically induced cells with or without dexamethasone (S_OB+D/S_OB-D. Representative results from three independent experiments are given. Mean frequencies calculated from three independent experiments are given in each histogram. **(B)** Percentages of CD83 and CD14 positive cells. **(C–E)** Median fluorescence intensities (MFI) of CD80, CD86, CD197, CD209, CD1a and HLA-DR expression. Data of three independent experiments are given as means ± SD and compared using one-way ANOVA followed by Tukey’s multiple comparisons tests (n=3, *p < 0.05, **p < 0.01, ***p < 0.001; ^#^p < 0.5 compared to CD14^+^ mo).

To analyze the phenotypic features of DC precursors and MoDC, surface markers were detected by flow cytometry. In pre-experiments, we compared the influence of 5- and 10-fold concentrated secretomes on DC maturation (as shown in the **Supplementary Figure 2**). Since no significant differences were observed between both groups, we decided to continue the experiments with 5-fold concentrated JPC secretomes. As illustrated in **Figure 2A** and **Table 3**, separated CD14^+^ population weekly expressed CD83, CD80, CD1a, CD209 and CD197, but they show strong CD86, CD14 and HLA-DR surface expression. Highly purified CD14^+^ monocytes (vitality of 97.4% ± 0.6%) were stimulated by DC induction cytokine cocktails. After 6 days, the number of CD14^+^ cells decreased to 1.7% ± 1.4%. The percentages of CD83, CD80, CD1a, CD209 and CD197 positive cells were 96.8% ± 2.8%, 98.7% ± 0.7%, 38.8% ± 40.7%, 96.8% ± 1.4% and 95.4% ± 1.4% respectively. Higher expressions of CD86 and HLA-DR were detected on the induced cells compared to their precursors (**Table 3**). Therefore, MoDCs maturation in this study was shown to be efficient. The addition of 5-fold and 10-fold concentrated JPCs secretomes to DCs during the maturation process had no effect on the viability of MoDCs (as shown in **Table 4**). Significantly decreased number of cells expressing CD83 and significantly increased number of cells expressing CD14 were detected in the S_OB+/-D DC groups compared to surface expression on DCs cultured without secretomes or under S_CO (from untreated JPCs) conditions (**Figure 2B**). MoDCs from the S_OB+/-D cell groups expressed significantly lower levels of CD80, CD86 and CD197, and significantly higher levels of CD209 in comparison to those detected under control conditions (without secretome) and S_CO conditions (**Figures 2C, D**). MoDCs generated under S_CO condition showed no significant effects on cell surface marker expression. We observed only little differences in induced effects on DC cell surface marker expression when treated with 5-fold and 10-fold secretomes (**Supplementary Figure 2**). Therefore, 5-fold concentrated secretomes were used for further experiments. The effect of 5-fold secretomes on CD1a and HLA-DR expression was further analyzed. S_OB+D decreased CD1a^+^ cells compared to number of others but not significantly, due to high donor variations (**Figure 2E**). There’s no significant difference in HLA-DR expression on cells generated under different conditions (**Figure 2E**).

**Table 3:** Efficiency of CD14^+^ monocyte-derived DC maturation.

Surface markers	Percentages of positive cells (%)		MFI	
	Day 0	Day 6	Day 0	Day 6
CD83	0.7 ± 0.6	96.8 ± 2.8 *	3.6 ± 16.8	36.9 ± 10.2
CD80	0.7 ± 0.7	98.7 ± 0.7 *	19.1 ± 5.2	78.4 ± 5.7 *
CD86	90.9 ± 2.8	98.9 ± 0.6	27.8 ± 4.6	527.2 ± 58.9 *
CD1a	6.6 ± 1.3	38.8 ± 40.7 *	55.3 ± 31.9	122.7 ± 183.5
CD209	2.7 ± 0.9	96.8 ± 1.4 *	39.6 ± 22.9	129.3 ± 30.8
CD197	1.8 ± 2.5	95.4 ± 1.4 *	35.4 ± 7.9	360.9 ± 95.8 *
CD14	97.4 ± 0.6	1.7 ± 1.4 *	69.5 ± 19.2	22.3 ± 11.4
HLA-DR	90.9 ± 0.9	99.6 ± 0.4	17.0 ± 6.5	80.9 ± 12.3 *

After separation of CD14^+^ cells from PBMCs, the population was analyzed for cell surface markers using FACS (day 0). Cells were further cultured in DC cytokine cocktail for 6 days and analyzed for cell surface markers on day 6. Data of three independent experiments are given as means ± SD and compared using student’s t tests (n=3, *p<0.05 compared to day 0).

**Table 4:** Percentages of viable DCs generated in the presence of JPC secretomes.

	Control		Addition of JPC secretomes		
		Concentration	S_CO	S_OB+D	S_OB-D
Percentage of viable cells (%)	94.47 ± 1.23	5-fold	96.76 ± 0.54	94.02 ± 1.54	93.51 ± 2.30
		10-fold	96.78 ± 1.01	93.26 ± 0.67	90.34 ± 3.20

The viability of DCs generated without (control) or in the presence of JPC secretomes (S_CO/S_OB+D/S_OB-D) was determined after propidium iodide staining by flow cytometry. The data of four independent experiments are shown as mean ± SD (n=4).

### 3.3 Effects of JPC secretomes on homotypic cluster formation of MoDCs

**Figure 3:**
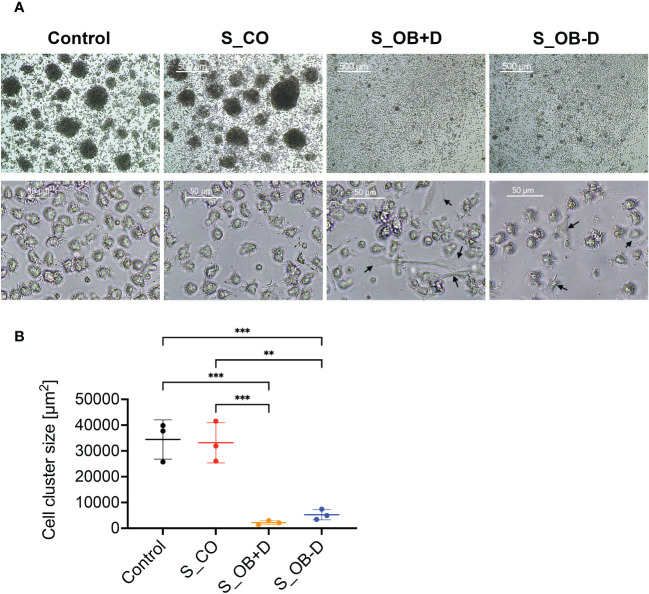
Effects of JPC secretomes on the homotypic cluster formation and the morphology of MoDCs. CD14^+^ monocytes were cultured with DC cocktails for 6 days (control). 5-fold concentrated secretomes of JPCs treated with control medium (S_CO), osteogenic medium with or without dexamethasone (S_OB+D/S_OB-D) were added to the DC induction cocktail respectively. **(A)** Representative microscopic images of three independent experiments (scale bars represent 500 and 50 µm respectively). Black arrows are pointing at adherent cells in non-round shape. **(B)** Quantification of cell cluster size using ImageJ. Results of three independent experiments are given as means ± SD and compared using one-way ANOVA followed by Tukey’s multiple comparisons tests (n=3, **p < 0.01, ***p < 0.001).

As shown in **Figure 3A**, large homotypic cell clusters were formed after culturing in DC differentiation cocktail (control) and under the influence of the cocktail additionally supplemented with the JPC secretome of undifferentiated JPCs (S_CO) for 6 days. Only a small number of aggregated cells were observed under the influence of the secretome from osteogenically differentiated JPCs, treated with or without dexamethasone (S_OB+D/S_OB-D). The quantification of the cell cluster size resulted in significantly decreased formation of large DC clusters under the influence of S_OB+D and S_OB-D secretomes. The smallest DC cluster size was detected in the S_OB+D group. However, differences to the S_OB-D did not reach significance and were just as little as between of the Control and S_CO groups (**Figure 3B**). In terms of cell shape, MoDCs showed a characteristic morphology in the Control and S_CO groups with numerous dendrites and a round and “tree-like” shape. Some adherent cells in non-round shape were observed in the S_OB+/-D groups (black arrows).

### 3.4 Effects of JPC secretomes on the phagocytic activity of MoDCs

**Figure 4:**
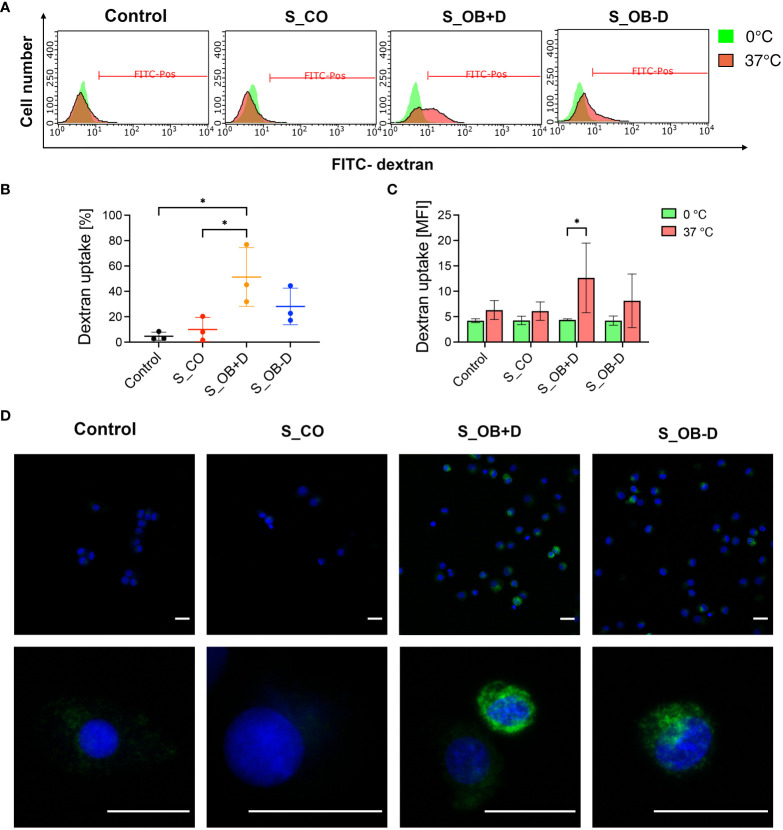
Effects of JPC secretomes on the dextran uptake ability of maturated MoDCs. DCs generated by indicated treatments (without secretomes or after the addition of 5-fold concentrated JPC secretomes) were incubated with 1 mg/mL FITC-dextran either on ice (0°C) or within an incubator (37°C) for 1 hour and analyzed by flow cytometry and fluorescence microscopy. **(A)** Representative FACS graphs of three independent experiments. **(B)** Percentage of dextran-positive DCs. **(C)** Mean fluorescence intensity (MFI) of dextran uptake by DCs on ice and at 37°C. Results of three independent experiments are given as means ± SD and compared using one-way ANOVA followed by Tukey’s multiple comparisons tests (n=3, *p<0.05). **(D)** Microscopic fluorescence images of FITC-dextran uptake (green) by DCs. Cell nuclei were stained by DAPI (blue). Scale bars represent 20 µm.

MoDCs generated without the addition of secretomes or in the presence of different JPC secretomes were compared in terms of their phagocytic activity by testing their ability for FITC-dextran uptake. S_OB+/-D developed MoDCs both showed phagocytic ability (**Figure 4A**). The percentage of dextran-positive cells was significantly increased in the S_OB+D compared to the Control and S_CO DC groups, whereas a clearly increased trend of dextran-positive cells was also detected in the S_OB-D DC group without reaching significance (**Figures 4B, C**). **Figure 4D** show representative microscopic images of green fluorescence FITC-dextran uptake mainly in DCs generated under the influence of secretomes from osteogenically induced JPCs.

### 3.5 Effects of JPC secretomes on the stimulatory ability of MoDCs towards lymphocytes/CD4^+^ T cells

**Figure 5:**
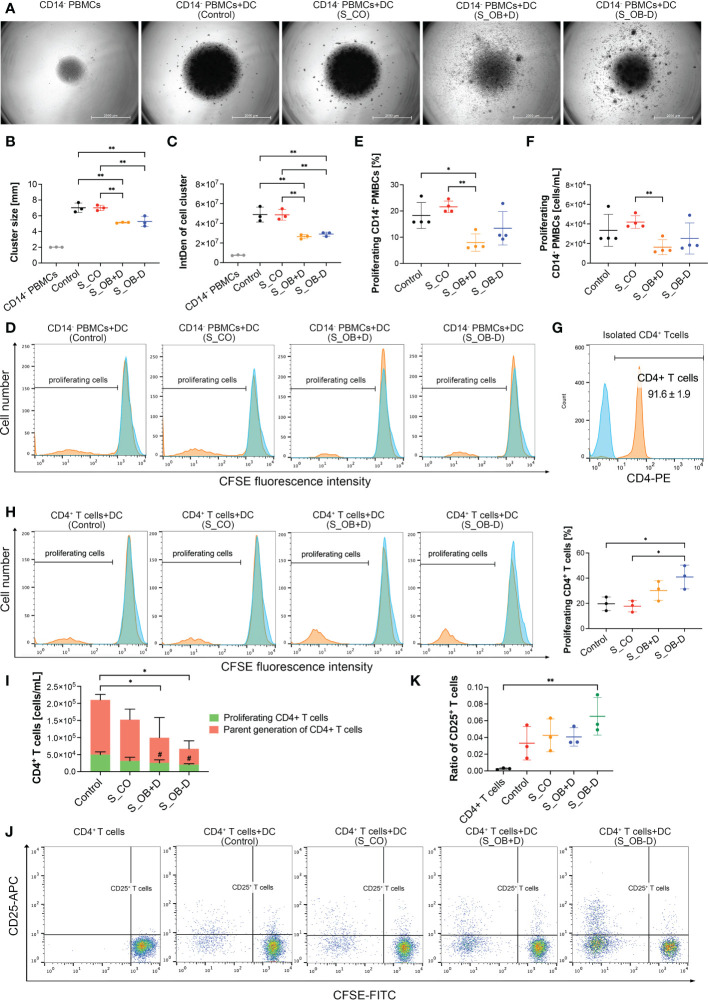
Effects of JPC secretomes on lymphocytes stimulatory ability of maturated MoDCs. CFSE-labeled CD14^-^ PBMCs **(A–F)** and CD4^+^ T cells **(G–K)** were cocultured (ratio 1:1) with MoDCs generated under different conditions (without secretomes (control) or in the presence of 5-fold concentrated JPC secretomes) for 3 days. Cell cluster size and density of the cocultured CD14^-^ PBMCs was observed by microscopy and quantified by ImageJ. **(A)** Representative microscopic images of three independent experiments (scale bars represent 2000 µm). Quantification of cell cluster size **(B)** and integrated density **(C)** by ImageJ. Data of three independent experiments are given as means ± SD and compared using one-way ANOVA followed by Tukey’s multiple comparison tests (n=3, *p < 0.05, **p < 0.01). **(D)** Representative histograms of three independent experiments of CD14^-^ PBMCs. Blue peaks represent monocultured CFSE-labeled CD14^-^ cells (unstimulated parent generation). Percentage **(E)** and concentration **(F)** of proliferating CD14^-^ PBMCs. Results of four independent experiments are given as means ± SD and compared using one-way ANOVA followed by Tukey’s multiple comparisons tests (n=4, *p < 0.05, **p < 0.01). **(G)** Purity of CD4^+^ T cells after isolation using the CD4^+^ T cell isolation kit (%). **(H)** Representative histograms of CD4^+^ T cell stimulatory experiments and percentage of proliferating CD4^+^ T cells. Blue peaks represent monocultured CFSE-labeled CD4^+^ T cells (unstimulated parent generation). **(I)** Concentration of CD4^+^ T cells after coculturing with differently generated MoDCs. Concentration of all CD4^+^ T cells and proliferating cells are given as means ± SD respectively and compared using one-way ANOVA followed by Tukey’s multiple comparison tests (^#^ means p<0.05 compared to the control group). **(J)** Representative dot blots for CD25 expression of monocultured CD4^+^ T cells or cocultures with differently generated DCs. **(K)** Ratio of CD25^+^ T cells within the whole CD4^+^ T cell population was calculated (CD25^+^ T cells/CD25^-^ T cells). **(G–K)** Data of three independent experiments are given as means ± SD and compared using one-way ANOVA followed by Tukey’s multiple comparison tests (n=3, *p < 0.05, **p < 0.01).

To compare the stimulatory ability of differently generated MoDCs on lymphocytes, they were cocultured with autologous CFSE-labeled CD14^-^ PBMCs for 3 days. After this incubation period, cells mainly gathered in the center of the well bottom under all cultivation conditions. However, cell distribution within the wells where CD14^-^ PBMCs were cocultured with DCs from S_OB+/-D conditions described a higher spreading beyond the well center, as shown in **Figure 5A**. The sizes and densities of formed cell clusters in the well center were shown to be significantly smaller/lower when CD14^-^ PBMCs were cocultured with MoDCs from the S_OB+/-D groups compared to those from the control and S_CO groups (**Figures 5B, C**), indicating fewer proliferating cells in these groups. This result was also confirmed by flow cytometry: significantly lower numbers of proliferating CD14^-^ PBMCs were detected in the coculture with S_OB+D DCs (**Figures 5D–F**), whereas the number of CD14^-^ PBMCs proliferating cells in S_OB-D coculture condition was also remarkably decreased, but without reaching significance (**Figures 5E, F**). Further, purified CD4^+^ T cells from CD14^-^ PBMCs (**Figure 5G**) were cocultured with differently generated MoDCs for 3 days. In contrast to the results of CD14^-^ PBMCs, significantly higher percentage of proliferating CD4^+^ T cells were detected in the S_OB+/-D groups. However, both the proportion of the whole CD4^+^ T cell population and that of proliferating CD4^+^ T cells significantly decreased in the S_OB+/-D groups (**Figure 5I**). The increased percentage of proliferating CD4^+^ T cells and observed decreased concentration of all CD4^+^ T cells in the S_OB+/-D groups indicated that CD4^+^ T cell numbers of parent generation were decreased probably due to cell apoptosis (**Figure 5H**). After CD25 labeling of the CD4^+^ cell population, we detected the highest ratio of regulatory CD4^+^/CD25^+^ T cells in the cocultures with MoDCs from the S_OB-D secretome group (**Figures 5J, K**).

### 3.6 Effects of JPC secretomes on gene expression of MoDCs

**Figure 6:**
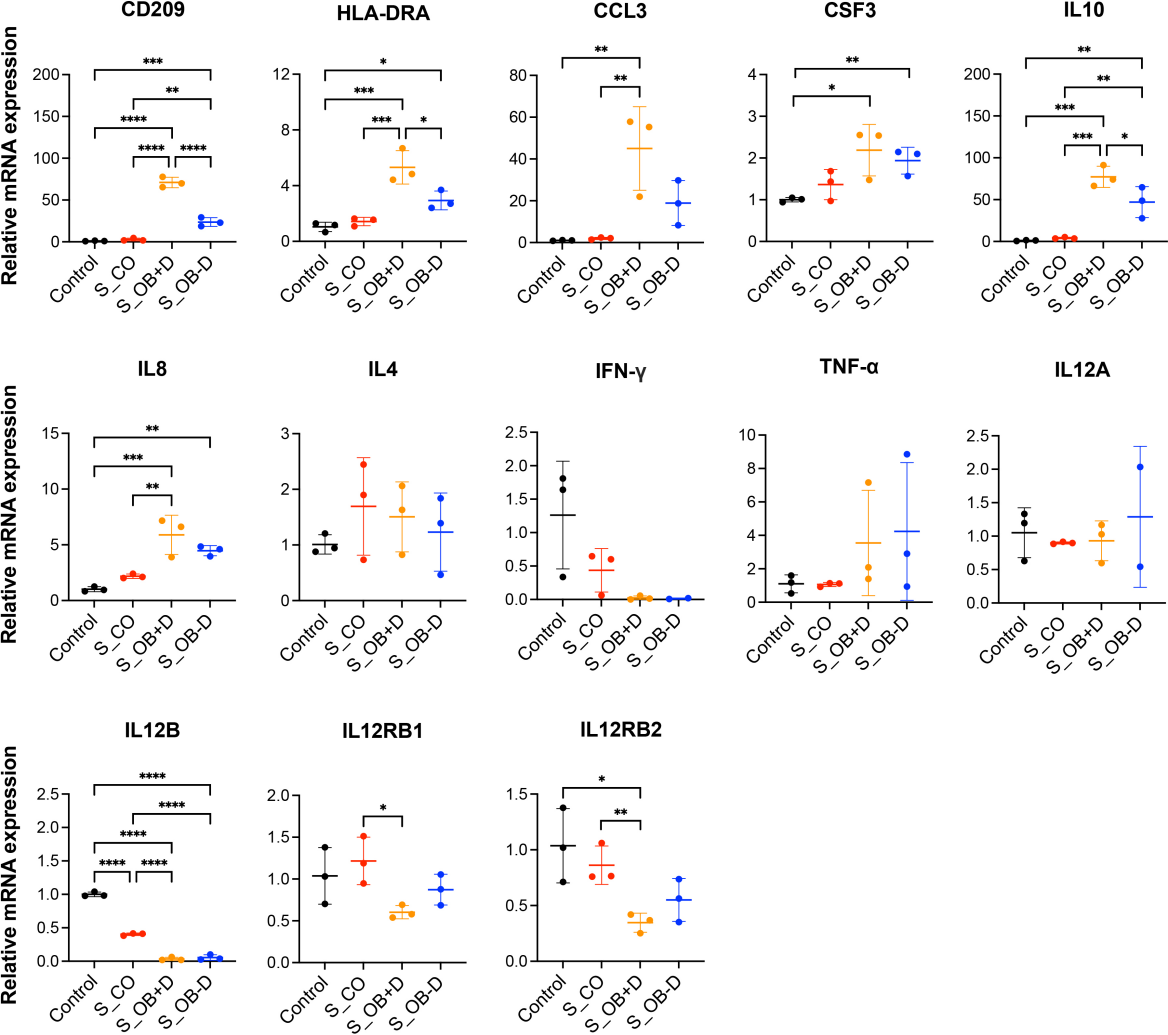
Effects of JPC secretomes on gene expression levels of maturated MoDCs. The relative gene expression levels of DCs generated by indicated treatments (control or after supplementation with 5-fold concentrated JPC secretomes) were presented as 2^-ΔΔCt^. Mean ± SD values of three independent experiments were calculated and compared using one-way ANOVA and Tuckey’s multiple comparison tests (n=3, *p < 0.05, **p < 0.01, ***p < 0.001, ****p < 0.0001).

The mRNA levels of MoDCs generated under the different conditions were quantified by real-time PCR. As shown in **Figure 6**, mRNA levels of CD209, HLA-DRA, CSF3 (G-CSF), IL10 and IL8 were significantly up-regulated in the S_OB+/-D DC cell groups compared to those detected in the control group without secretomes (control). CCL3 (MIP-1α) gene expression levels were significantly higher in the S_OB+D but not in the S_OB-D DC group compared to those detected in the control and S_CO cell groups. No significant differences of IL4 and TNF- α levels were detected. MoDCs differentiated under S_OB+/-D conditions expressed extremely low or undetectable IFN- γ levels. Generated MoDCs expressed under all conditions extremely low levels of IL12A (IL-12p35), but they expressed IL12B (IL-12p40). IL12B mRNA levels were significantly reduced in MoDCs which were supplemented with the JPC secretome from untreated and osteogenically induced cells (S_CO, S_OB+/-D). Levels of IL-12 receptor (IL-12Rβ1/IL-12Rβ2) were detected to be significantly reduced only in MoDCs from the S_OB+D cell group in comparison to those detected in the S_CO group. IL-12Rβ2 gene expression showed in the tendency also a clear downregulation, without reaching significance.

### 3.7 Effects of JPC secretomes on the secretory function of maturated DCs

**Figure 7:**
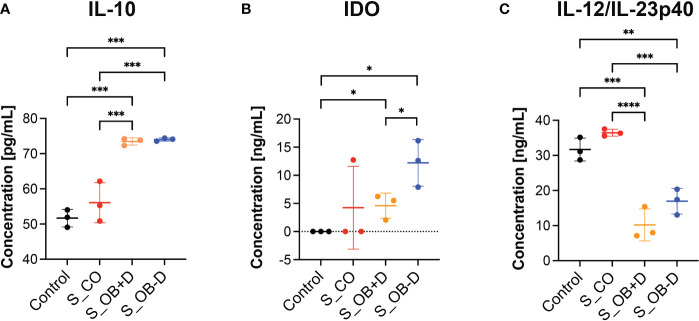
Effects of JPC secretomes on protein release by maturated MoDCs. **(A–C)** IL-10, IDO and IL-12/IL-23p40 levels in supernatants from DCs generated under the indicated treatments (control – without secretome or after the addition of 5-fold concentrated JPC secretomes). Quantification was performed using specific ELISAs. Mean ± SD values of three independent experiments were calculated and compared using multiple t tests (n= 3, *p < 0.05, **p < 0.01, ***p < 0.001, ****p < 0.0001).

To detect key factors of immunosuppression and/or inflammation released by MoDCs generated without or in the presence of secretomes, supernatants of MoDCs and JPC secretomes were analyzed by specific ELISAs. IL-10, IDO or IL-12/IL-23p40 were undetectable in JPC secretomes. As shown in **Figure 7**, MoDCs generated under S_OB+/-D conditions significantly up-regulated IL-10 protein expression (**Figure 7A**) and suppressed their IL-12/IL-23p40 release (**Figure 7C**), consistent with the results obtained by quantitative gene expression analysis. Additionally, significantly increased levels of indoleamine 2,3-dioxygenase 1 (IDO) were detected under S_OB+/-D in comparison to control culture conditions, levels under the S_OB-D condition were shown to be significantly higher than IDO levels under the S_OB+D condition (**Figure 7B**).

## 4 Discussion

Originating from experiments focusing on the immunomodulatory function of bone marrow MSCs, considerable knowledge regarding MSC-mediated inhibition of DC maturation and activation has been gained (28–31). For jaw bone regeneration, we are working with MSCs derived from the jaw periosteum tissue. In previous studies we were able to confirm that two- and three-dimensionally cultured JPCs effectively suppressed monocyte-derived DC maturation in a transwell coculture system (6, 7). As stem-cell free therapies are easier to transfer from bench site into the clinical routine, we investigated the effects of secretomes from undifferentiated and osteogenically differentiated JPCs on the maturation and function of MoDCs. For DC generation, a protocol different from the original one used for our previous studies was chosen starting from the isolation of CD14^+^ monocytes. Using this highly purified CD14^+^ cell fraction (97.4% ± 0.6%), generated MoDCs aggregated and formed large homotypic clusters while MoDCs supplemented with secretomes from osteogenically induced JPCs (S_OB+/-D) were reduced in their capability of cluster formation (**Figure 3A**). Termeer and colleagues showed evidence that DCs are able to form cell clusters, and reveal characteristics of mature DCs after isolation from formed clusters with high expression of MHC class II, CD80 and CD86 (32). Kubo and co-authors report on DC cluster formation after LPS stimulation, which could be inhibited by tofacitinib, a JAK (Janus kinase) inhibitor resulting in reduced CD80/CD86 surface expression on DCs (33). Homotypic clustering of DCs seems to be closely correlated with the maturation state of DCs and might facilitate intercellular signal transduction or even an exchange of antigenic material between APCs (34, 35). In addition to the reduced capacity of cluster formation, JPC secretomes from osteogenically induced cells significantly down-regulated the expression of B7-costimulatory molecules (CD80/CD86), C-C chemokine receptor (CD197) on maturating MoDCs (**Figures 2C, D** and **Supplementary Figures 2C–E**) and decreased the number of CD83 expressing MoDCs (**Figure 2B** and **Supplementary Figure 2A**). CD83 represents an activation marker for antigen presenting cells (36). The same secretomes were able to increase the number of CD14^+^ cells (**Figure 2B** and **Supplementary Figure 2B**). This marker is specific for immature monocytes (37). Secretomes from osteogenically induced JPCs in the absence of dexamethasone showed similar but in tendency lower effects than those from dexamethasone-treated JPCs (**Figures 2B–D** and **Supplementary Figures 2A–E**). These results indicated that osteogenically differentiated JPC secretomes inhibit phenotypic maturation of MoDCs.

Surprisingly, HLA-DR surface expression was not affected by JPC secretomes although mRNA levels were significantly up-regulated (**Figure 2E** and **Figure 6**). These opposite results can be explained by the theory that immature DCs constitutively present self-peptides bound to MHC II molecules, but these complexes are degrading quickly and therefore a more transient expression on the cell surface is observed compared to that on mature DCs (38). Therefore, up-regulation of HLA-DRA mRNA but not up-regulated expression of HLA-DR on the surface of MoDCs supplemented with OB +/- dexamethasone secretomes suggested that these MoDCs were less mature than MoDCs of the control and S_CO groups. Further, there is the possibility that only a DC subset responds to the JPC secretome treatment. This issue should be analyzed in future studies.

CD209 is a cell surface C-type lectin expressed on DCs involved in cell-cell interactions through its capacity to bind ICAM-3 and ICAM-2 (39, 40). CD209 surface expression is usually upregulated in particular on immature DCs (41, 42). In our study, CD209 surface expression was strongly induced after the DC maturation. We detected a significant increase in CD209 surface expression (**Figure 2D** and **Supplementary Figure 2F**) and mRNA expression (**Figure 6**) under the influence of secretomes from osteogenically induced JPCs. Since CD209 is known to be an antigen-uptake receptor for pathogens (41, 43–45), we analyzed the phagocytic ability of MoDCs generated under the different conditions. The same secretome (from dexamethasone-treated JPCs) which led to significantly upregulated CD209 gene expression, simultaneously induced the highest dextran-uptake in MoDCs (**Figure 4B**).

Mature DCs lose their antigen-capturing capacity, but they obtain a T cell stimulatory function (23). The coculture experiments performed in our study gave evidence for significant lower cluster formation, density and proliferating CD14^-^ PBMCs in the presence of JPC secretomes from osteogenically treated JPCs, whereas the highest effect came from secretomes of dexamethasone-treated JPCs (**Figures 5D–F**). When CD4^+^ T cells were applied in the coculture experiment, MoDCs generated with secretomes from osteogenically treated JPCs significantly reduced the number of proliferating CD4^+^ T cells and the number of the parental CD4^+^ T cells (**Figure 5I**). CD80/CD86 co-stimulatory involvement for optimal T cell activation was firstly described by Azuma and co-authors (46, 47). Blockage of CD80 and CD86 on MoDCs led to a decreased capacity in activation of naive CD4^+^ T cells (48). This could be at least partially the underlying mechanism by which JPC secretomes are able to elicit the observed reduced CD4^+^ T cell stimulatory function.

In addition, interactions between DCs and helper T (Th) cells during antigen presentation determine the Th cell differentiation fate (49). Apart from direct cell-to-cell interactions by costimulatory molecules, mature DCs also exert T cell stimulatory functions by the secretion of pro-/anti-inflammatory cytokines. IL-12p40 is a component of IL-12p70 and IL-23 and a cytokine activating T cells (50). Previous studies have reported that IFN-γ (51) and IL-12 (52, 53) drive the pro-inflammatory Th1 transcriptional program. DC-derived IL-12p40 is required for the activation and maintenance of IFN-γ-producing Th1 cells (52, 54). In our study, DC cocktail supplementation with secretomes from osteogenically induced JPCs led to significant down-regulated IL12B mRNA levels (**Figure 6**) and a significant down-regulation of IL-12p40 release by MoDCs (**Figure 7C**). IFN-γ mRNA levels were almost completely abolished in the presence of the secretome from osteogenically induced JPCs. These results indicate that JPC secretomes are potentially able to suppress Th1 cell polarization function of MoDCs by an effective down-regulation of the pro-inflammatory cytokines IFN-γ and IL-12p40.

IDO is an enzyme capable to degrade the essential amino acid tryptophan, participating in regulation of T cell immunity by the tryptophan metabolic pathway (55). Numerous studies report on the promotion of T cell tolerance and suppression of T cell responses by IDO expressing cells (56). In our study, the secretome derived from osteogenically induced JPCs was able to significantly up-regulate IDO expression in MoDCs, and the secretome from osteogenically induced JPCs in the absence of dexamethasone seemed to be the most powerful supplement in inducing IDO up-regulation (**Figure 7B**). Apart from this, the addition of the same secretomes significantly induced the up-regulation of mRNA and protein levels of the anti-inflammatory cytokine IL-10 (**Figure 6** and **Figure 7A**). IL-10 is known to inhibit T cell proliferation and to initiate the development of regulatory T cells limiting the development of Th1 or Th2 effector cells (57, 58). The coculture experiments performed in this study revealed that DCs generated with secretomes from osteogenically induced JPCs significantly suppressed proliferation of CD4^+^ T cells and potentially induce CD4^+^ T cell apoptosis (**Figure 5I**). MoDCs generated with secretome from osteogenically induced JPCs in the absence of dexamethasone significantly increased the number of regulatory CD25^+^ T cells within the CD4^+^ T cell population (**Figure 5K**). These evidences revealed that MoDCs generated in the presence of secretomes from osteogenically induced JPCs without dexamethasone seemed to act as tolerogenic DCs and induced expansion of regulatory T cells. This finding indicates that JPC secretomes, especially those of osteogenically stimulated cells without dexamethasone could be able to suppress immune responses and induce tolerance after implantations of cell-free engineered constructs.

It has been reported that MIP-1α (macrophage inflammatory protein-1 or CCL3) is not active on mature but immature DCs, mediating their migration into peripheral sites (59). In our experiments, we detected a significant upregulation of MIP-1α gene expression in DCs supplemented with JPC secretomes, in particular with those from dexamethasone-treated JPCs. Further MoDC gene expression of other chemokines such as G-CSF (CSF3) and IL-8 were shown to be significantly up-regulated, indicating that JPC secretomes were not only able to up-regulate expression or release of anti-inflammatory cytokines (IL-10) and to downregulate the secretion of pro-inflammatory cytokines (IL-12, IFN-γ, MIP-1α), but also to enhance the production of chemokines.

Strategies were developed for the use of DCs as “positive vaccines” for anti-tumor therapeutic approaches whereby monocytes are isolated from the peripheral blood of the patient, loaded with tumor antigens, and subsequently matured. The activated DCs are then re-infused into the patient where they migrate to the lymph nodes to interact with naïve T cells in order to induce the activation of effector T cells (60). Another therapeutic option is to apply tolerogenic DCs as “negative vaccines” in order to induce tolerance against transplanted tissues (61). In our study, we could demonstrate that osteogenic JPC secretomes activate the generation of tolerogenic DCs. This finding indicates that TE constructs colonized with JPCs will probably not induce an inflammatory immune reaction. On the other site, it is conceivable that tolerogenic DCs can be applied together with TE grafts in order to ensure the prevention of immune reactions and to facilitate a favorable integration of the implants into the surrounding tissue.

Taken together, in the present study we examined for the first time the effects emanating from different JPC secretomes on DC maturation of CD14^+^ monocytes. JPC secretomes seemed to be sufficient to efficiently inhibit MoDC maturation and this ability is linked to their osteogenic differentiation state. Interestingly, JPC secretomes from untreated cells had little to no effect on maturation of MoDCs, and secretomes derived from dexamethasone-treated cells showed stronger effects compared to those from cells osteogenically stimulated in the absence of dexamethasone. Since it is well-known that dexamethasone elicits immunosuppressive effects, it is important to note that the obtained results are not mediated by dexamethasone contained in the culture medium/secretomes. Before secretome collection, dexamethasone dissolved in the culture medium was removed and JPCs were incubated with the basal medium only for the last 24 hours (as mentioned in the section 2.2 of materials and methods).

## 5 Conclusion

**Figure 8:**
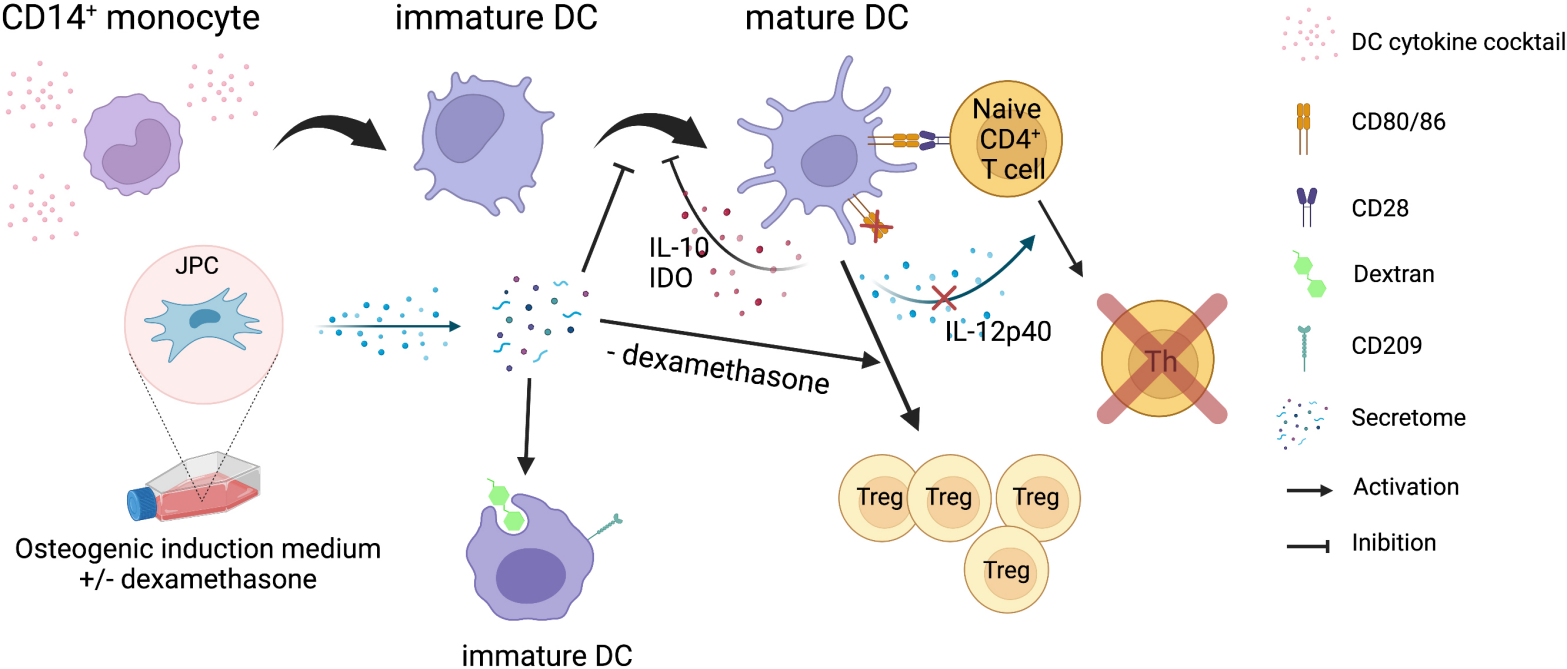
Summary of the results obtained in the present study. Illustration of the effects of JPC secretomes derived from osteogenically differentiated cells on MoDC maturation and function. Secretomes from osteogenically induced JPCs inhibit the maturation of CD14^+^ monocyte-derived DCs and their CD4^+^ T cell stimulation function, but promote phagocytic function of immature DCs. Secretome from osteogenically induced JPCs in the absence of dexamethasone induced expansion of regulatory T cells. The illustration was created with BioRender.

JPC secretomes from osteogenically induced cells are able to inhibit phenotypic maturation of MoDCs by destabilizing cluster formation and down-regulation of co-stimulatory surface markers. Generated MoDCs show enhanced antigen uptake ability and suppressed CD4^+^ T cell stimulatory function displaying a more immature phenotype. MoDCs generated in the presence of JPC secretomes from osteogenically induced cells without dexamethasone were able to promote regulatory CD25^+^ T cell expansion. The underlaying mechanism probably involves the up-regulation of IL-10 and IDO, the down-regulation of the co-stimulatory molecules CD80/86 and the down-regulation of the pro-inflammatory IL-12p40 (**Figure 8**). These findings indicate that TE constructs colonized with JPCs will probably not induce an inflammatory immune reaction after implantation. In order to ensure the prevention of immune reactions and to facilitate a favorable integration of the implants into the surrounding bone tissue, it is conceivable that tolerogenic DCs generated in the presence of osteogenic JPC secretomes will be applied together with TE grafts. The present study contributes to a better understanding of JPCs’ paracrine activity in order to optimize bone regenerative strategies using this cell type.

## Data availability statement

The original contributions presented in the study are included in the article/**Supplementary Material**. Further inquiries can be directed to the corresponding author.

## Ethics statement

The studies involving human participants were reviewed and approved by Ethics Committee of the Medical Faculty Tübingen. The patients/participants provided their written informed consent to participate in this study.

## Author contributions

WC contributed to the experiment design, performed the experiments, analyzed the data, wrote the first draft of the manuscript and contributed in editing the final draft of the article. FU and AS contributed to the methodology. SR contributed to administration issues and provided the resources. DA contributed to the experiment design, supervision and in editing the final draft of the article. All authors contributed to the article and approved the submitted version.
