# Supplementary material for: Secretomes derived from osteogenically differentiated jaw periosteal cells inhibit phenotypic and functional maturation of CD14+ monocyte-derived dendritic cells
Source: Front Immunol. 2023 Jan 9;13:1024509. doi: 10.3389/fimmu.2022.1024509 (PMC9868599; doi:10.3389/fimmu.2022.1024509)
Supplement: Supplementary file 1 [file DataSheet_1.docx]

Supplementary Material


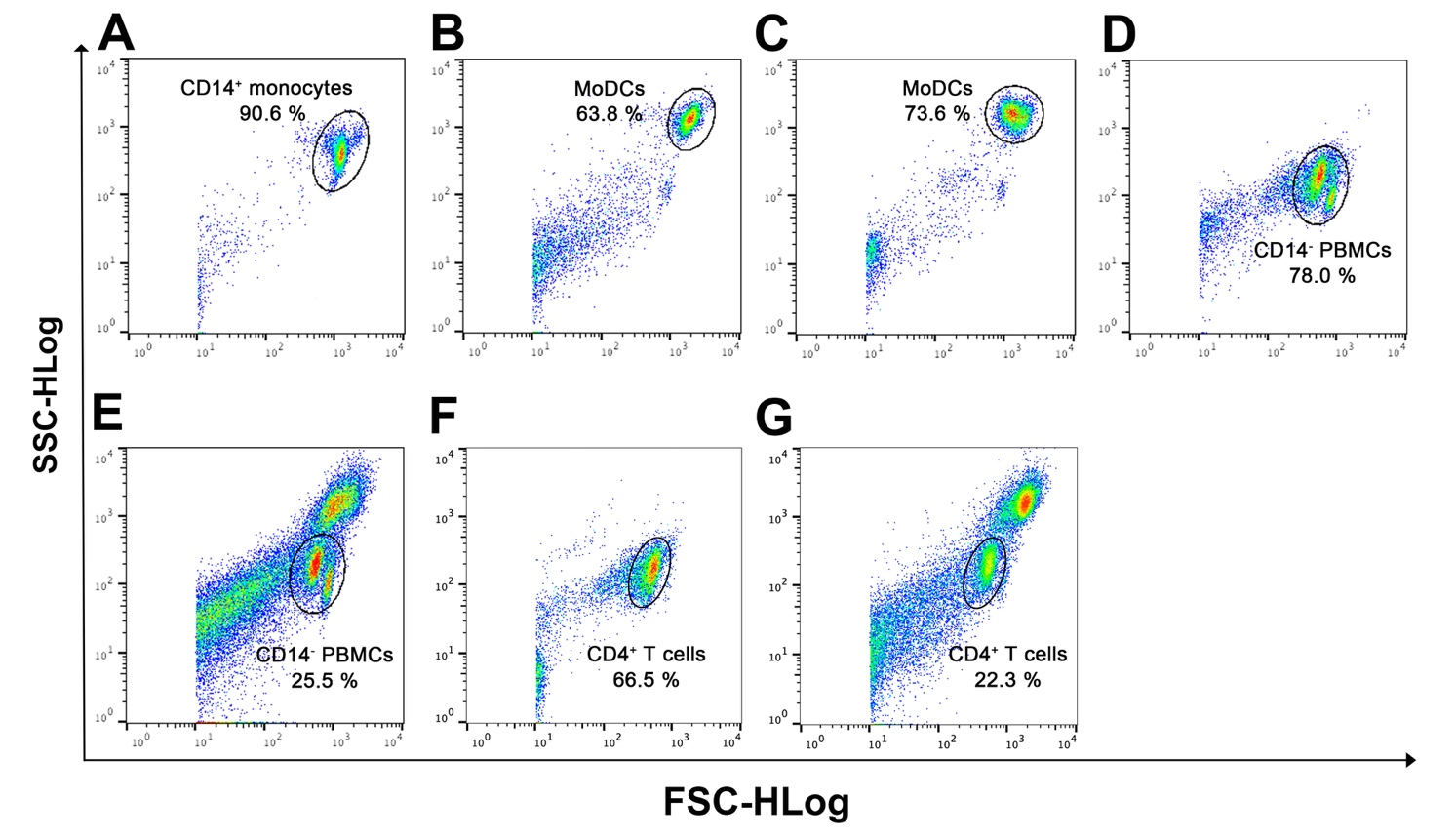


**Supplementary Figure 1.** Representative gating strategy for flow cytometry analyses. All analyzed cells were gated according to their size and granularity (forward scatter, FSC, cell size vs. side scatter, SSC, granularity) within the marked gate. (A) Dotplot of FSC vs. SSC-HLog for cell surface marker detection of CD14^+^ monocytes. (B) Dotplot of FSC vs. SSC-HLog for cell surface markers detection of dendritic cells (DCs). (C) Dotplot of FSC vs. SSC-HLog for analysis of the phagocytic activity of DCs. (D-G) Dotplot of FSC vs. SSC-HLog for mixed lymphocyte reactions. (D) Gating of monocultured CD14^-^ PBMCs. (E) Gating of CD14^-^ PBMCs cocultured with DCs. (F) Gating of monocultured CD4^+^ T cells. (G) Gating of CD4^+^ T cells cocultured with DCs. All percentages, mean fluorescent intensities and statistical analysis were performed inside the gate, excluding cell debris.


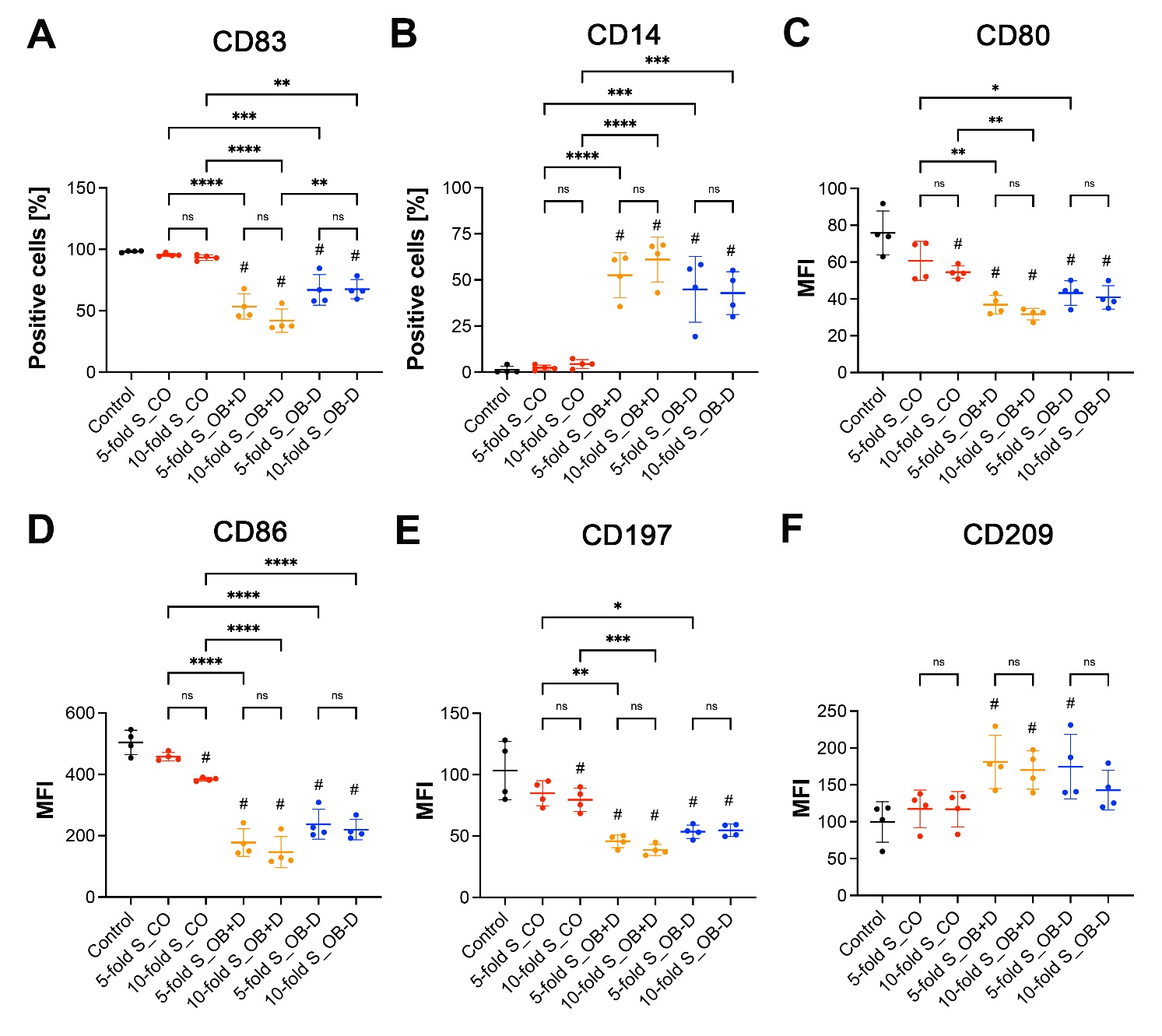


**Supplementary Figure 2.** Flow cytometry analysis (FACS) of cell surface markers on DCs generated without (control) or in the presence of 5 and 10-fold concentrated secretomes derived from JPCs treated with control medium (S_CO), osteogenic medium with or without dexamethasone (S_OB+D/S_OB-D). (A, B) Percentages of CD83 and CD14 positive cells. (C-F) Mean fluorescence intensity (MFI) of CD80, CD86, CD197 and CD209 surface expression. Data of four independent experiments are given as means ± SD and compared using one-way ANOVA followed by Tukey’s multiple comparisons tests (n=4, ^ns^p>0.05, *p<0.05, **p<0.01, ***p<0.001, ****p<0.001; ^#^ p< 0.5 compared to the group Control).

Supplementary Table 1. Information in terms of age and gender of included PBMCs donors. PBMCs for the generation of monocyte-derived dendritic cells differentiation were obtained from healthy donors.

|  | Age | Gender |
| --- | --- | --- |
| Donor 1 | 26 | male |
| Donor 2 | 32 | female |
| Donor 3 | 31 | male |
| Donor 4 | 29 | female |
